# The role of elective neck dissection in parotid acinic cell carcinoma: a systematic review

**DOI:** 10.1007/s10006-026-01633-6

**Published:** 2026-08-26

**Authors:** Sai Pabbati, Daniel Cook, James Heaton, Samuel Freeman, Manavaroa Taripo, Danilo Carluccio, Omar Breik

**Affiliations:** 1 Rural Clinical School, University of Queensland, 273 Bourbong St, Bundaberg West, Queensland Australia; 2 Oral and Maxillofacial Unit, The Royal Brisbane and Women’s Hospital, Brisbane, Queensland Australia

**Keywords:** Salivary gland tumours, Neck dissection, Parotid neoplasms, Acinic cell carcinoma

## Abstract

**Purpose:**

Acinic cell carcinoma (AciCC) is a rare parotid malignancy treated by parotidectomy with or without a neck dissection depending on clinical or radiographic neck node involvement. The role of elective neck dissection (END) in the clinically node-negative neck, intended to address occult lymph node metastases (OLNM), remains contentious.

**Methods:**

This review was conducted per PRISMA 2020. Searches through PubMed, EMBASE, and CINAHL were conducted up to May 2025. Data extraction focused on survival, recurrence outcomes and OLNM. Risk of bias was assessed using the Newcastle-Ottawa Scale.

**Results:**

From 741 screened articles, 13 retrospective cohort studies were included. Across 2,646 parotid AciCC cases, 788 underwent END, 77 demonstrating OLNM. Marked heterogeneity was observed among studies, with reported OLNM rates ranging from 0% to 37.5% and a weighted mean of 9.8%. High-grade transformation was associated with poorer survival; advanced T-stage showed a less consistent association, and grade-stratified nodal differences were generally based on small samples and not statistically significant.

**Conclusion:**

Routine END is not supported in low-grade, early-stage AciCC. High-grade transformation is an adverse prognostic factor; the independent value of advanced T-stage is less certain, resting largely on some single cohort studies. END has not been shown to improve survival, and comparisons are confounded by indication. Any role in higher-risk disease remains uncertain and should be individualised rather than routine. Multi-institutional registries with standardised reporting are needed to strengthen the evidence.

**Supplementary Information:**

The online version contains supplementary material available at 10.1007/s10006-026-01633-6.

## Introduction

 Acinic cell carcinoma (AciCC) is a rare malignant epithelial tumour of the salivary glands, primarily affecting the parotid gland [1]. While the prognosis for AciCC is generally favourable, optimal surgical management, particularly concerning the utility of elective neck dissection, remains contentious [2, 3]. Elective neck dissection (END) is an invasive surgical procedure, typically performed in addition to primary tumour removal, that involves cervical lymph node dissection in the context of no clinical or radiographic nodal involvement [2–4]. It aims to address potential occult lymph node metastases (OLNM) proactively. Currently, the standard treatment for AciCC involves surgical excision of the primary tumour, typically a total or partial parotidectomy [5]. However, uncertainty persists regarding the necessity and benefits of neck dissection in patients [2–4]. This uncertainty is reflected in the variability of clinical practice, where some institutions favour conservative local excision. In contrast, others advocate for routine END due to concerns over OLNM and potential impact on nodal recurrence and/or long-term survival.

Current literature reveals significant variability in reported rates of OLNM among cN0 patients, complicating evidence-based decision-making [3, 4]. Moreover, existing research predominantly consists of retrospective, single-institution studies with small sample sizes, limiting their capacity to yield definitive conclusions or inform standardised clinical protocols. The relatively indolent nature of AciCC, characterised by slow progression and low metastatic potential, further complicates the risk-benefit assessment of aggressive surgical strategies [2–5].

Thus, a notable knowledge gap exists due to the absence of robust, high-quality, large-scale evidence specifically addressing the role of END in managing cN0 parotid AciCC. This paper aims to bridge this gap through a systematic review of the existing literature, assessing the impact of END on OLNM detection, survival outcomes, recurrence rates, postoperative morbidity, and patient quality of life.

## Methods

This review was conducted in accordance with the PRISMA 2020 guidelines [6]. Ethics approval was not required, as this is a systematic review of previously published literature. The protocol for this review is registered under the International Prospective Register of Systematic Reviews (PROSPERO) (ID: CRD420251046849). There is currently no systematic review that sufficiently and specifically evaluates the impact of END in the context of cN0 parotid AciCCs. Eligible studies included randomised controlled trials, controlled clinical trials, and prospective or retrospective cohort studies. The included populations comprised patients of any age or gender diagnosed with parotid AciCC, irrespective of tumour stage or grade. The intervention of interest was END performed for cN0 parotid AciCCs, and studies were required to report END-related outcomes such as overall survival (OS), disease-specific survival (DSS), recurrence-free survival (RFS), or OLNM rates. The specific sample size of ENDs performed had to exceed five. Variations in the technique or extent of END were also considered eligible. Comparator groups included patients managed with any form of parotidectomy (superficial, total, or partial), therapeutic neck dissection (TND), observation, or other oncological treatments not involving END. Only peer-reviewed, English-language studies published from 1974 onwards were included. Studies were excluded if they were case reports, case series, in-vitro studies, cross-sectional studies, editorials, or other non-peer-reviewed literature. Research focusing exclusively on recurrent or metastatic AciCC, or studies with mixed salivary histology that did not report separate data for AciCC, were also excluded. In addition, studies comparing surgical techniques or adjuvant therapies without END-specific subgroup analysis were also excluded.

A comprehensive search of PubMed (MEDLINE), EMBASE, and CINAHL was conducted to identify relevant studies. The search strategy was developed in consultation with a university librarian and refined by the research team. Searches were completed by May 7, 2025, and results were managed using Covidence. The full electronic search strategies for all databases are provided in Supplementary Material 1.

An initial exploratory search was performed to identify common keywords and index terms from titles, abstracts, and reference lists. These terms were used to construct a refined search strategy. Using Covidence, title and abstract screening was conducted independently by two reviewers, followed by full-text screening of eligible studies. Discrepancies were resolved by a third reviewer. Reference lists of included articles were also reviewed to identify additional studies. Final inclusion was confirmed collaboratively by the review team.

A standardised data extraction template was piloted and reviewed by the research team. Data were extracted independently by multiple reviewers, with results discussed and refined through an iterative process to ensure consistency and completeness.

For each study, data extraction was conducted in accordance with the finalised protocol, with all variables entered as free text to accommodate the heterogeneity of reporting across studies. Extracted variables included study author, publication year, study design, sample size of parotid AciCC cases, number of patients undergoing END, and rates of OLNM. Where available, details of comparator groups (e.g. patients without END or with TND) were also recorded. In addition to study-level characteristics, outcome data relating to the effect of END, T-stage, and histological grade on overall survival, disease-specific survival, recurrence-free survival, disease-free survival (DFS), and OLNM rates were extracted. Where studies did not report AciCC-specific outcomes, data were noted at the broader cohort level with explicit annotation of this limitation. Studies for which raw data were available were summarised in accordance with the original reporting, without additional survival analysis. This approach prioritised accurate representation of published results and aimed to identify both existing evidence and significant gaps in outcome reporting. Extracted data were summarised narratively and presented in tabular form for clarity. Given the marked clinical and methodological heterogeneity, no meta-analysis was undertaken, and outcomes were synthesised narratively in accordance with the Synthesis Without Meta-analysis (SWiM) reporting guideline [7].

A methodological quality assessment of all included studies was conducted using the Newcastle–Ottawa Scale (NOS) for observational cohort studies [8]. The NOS evaluates three domains: selection of study cohorts (maximum 4 stars), comparability of cohorts based on design or analysis (maximum 2 stars), and assessment of outcomes and adequacy of follow-up (maximum 3 stars), with a maximum overall score of 9. Studies were then categorised as ‘good quality’ (3–4 stars in selection, 1–2 in comparability, and 2–3 in outcome), ‘fair quality’ (2 in selection, 1–2 in comparability, and 2–3 in outcome), or ‘poor quality’ (0–1 in selection, 0 in comparability, or 0–1 in outcome). Two reviewers independently applied the NOS, and discrepancies were resolved by a third reviewer.

Portions of data extraction and manuscript drafting were assisted by ChatGPT (GPT-5, OpenAI) and Claude (Opus 4.8, Anthropic). These models were used to refine phrasing, summarise extracted data, and check for consistency in reporting across included studies. All outputs were reviewed and verified by the authors for accuracy and intellectual content before inclusion.

## Results

**Fig. 1:**
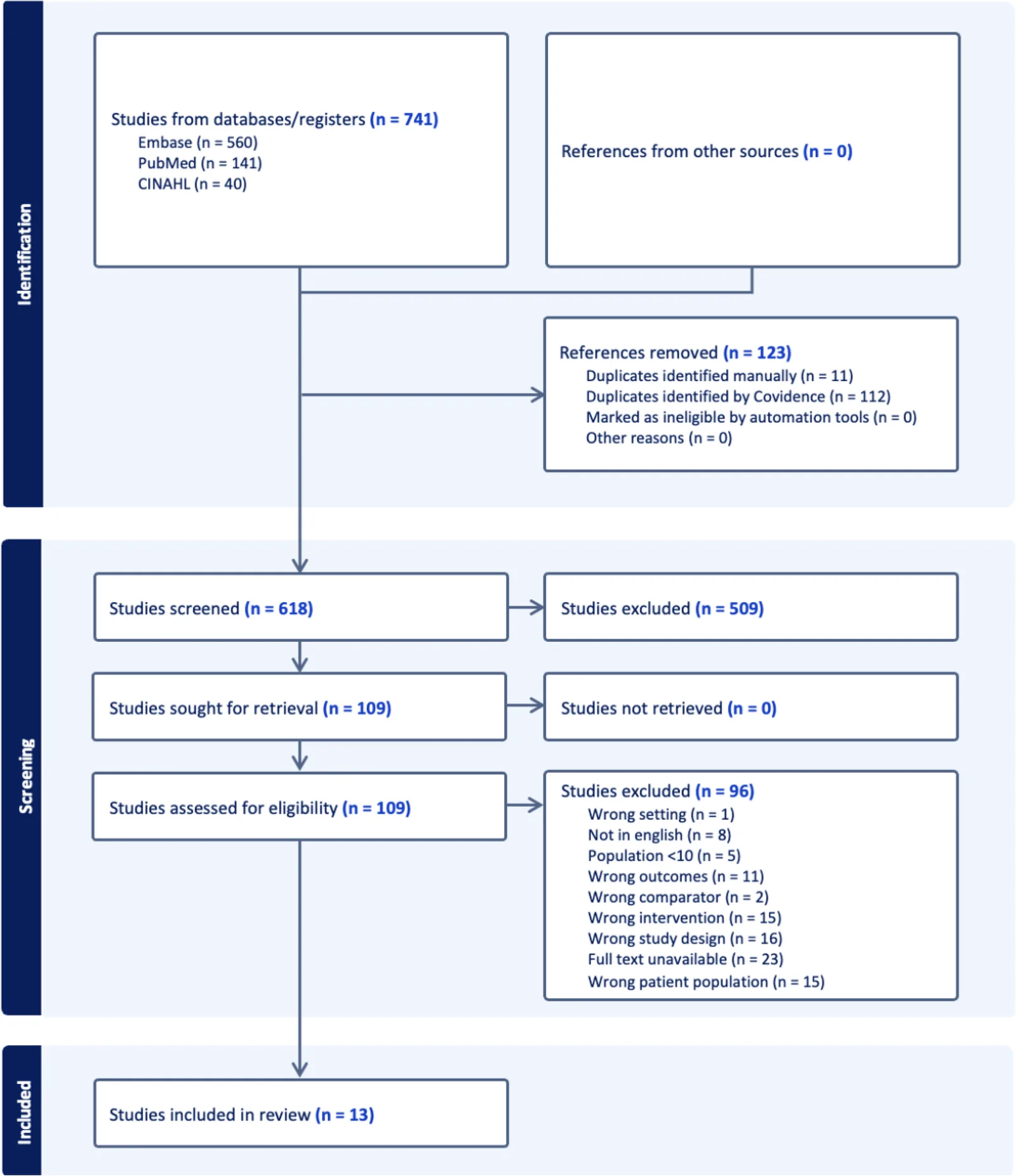
PRISMA flow chart of literature search [6]

Figure 1 summarises the literature search process for this review as per PRISMA 2020 guidelines [6]. The initial search of bibliographic databases identified 741 records. After the removal of 123 duplicates, 618 unique articles underwent screening. Title and abstract screening excluded 509 articles, and full-text review excluded an additional 96, primarily due to ineligible population, intervention, or insufficient reporting of outcomes. Thirteen studies published between 1993 and 2025 satisfied the inclusion criteria, all of which were retrospective cohort designs based on single- or multi-centre datasets.

Table 1 summarises the study characteristics, including author, publication year, study location, design, study period, total number of reported malignancies and ENDs, parotid AciCC cases and END, reported OLNM, and comparator group details if available. Table 2 summarises available data on the effect of T-stage, histological grade, and END on survival and OLNM rates in AciCC. It also serves to visualise which studies pooled OLNM rates with tumour stage, grade and survival outcomes. The role of END in improving survival and recurrence outcomes for AciCC remains unclear, with outcome reporting inconsistent across the included studies. Regarding overall survival, Fussey et al. [9] reported slightly higher OS in patients treated with local excision for AciCC without END (95%) compared to those who underwent END (90%). Grasl et al. [4] studied a parotid AciCC only population, and directly compared END with no neck dissection, and found no significant survival benefit (5-year OS 97% vs. 92%, *p* = 0.795; DSS 100% vs. 96%, *p* = 0.307; DFS 96% vs. 87%, *p* = 0.312). Dell’Aversana Orabona et al. [10] found no difference, with 100% OS in both END and non-END groups, although their small sample size and pooling of various parotid malignancy subtypes limit AciCC-specific interpretation [10]. Other studies that pooled histology also reported no statistically significant effect of END on OS [11, 12].

**Table 1:** Study characteristics

Study	Location	Study Design	Total years	Data collection (start-end)	Total sample size	Total ENDs	Parotid AciCCs	Parotid AciCC ENDs	pN+	Comparator Group
Ning, 2025	Beijing, CN	Retrospective	10 years	2012–2022	427	75	9	9	2/9	END vs. no-END
Vedula, 2023	Newark, US	Retrospective	5 years	2010–2015	9405	3396	2125	570	42/570	END vs. no-END
Kapsalas, 2023	Erlangen, DE	Retrospective	15 years	2007–2022	94	94	47	47	5/47	No comparator
van Weert, 2022	Amsterdam, NL	Retrospective	37 years	1979–2016	89	10	76	10	0/10	END vs. MRND
Fussey, 2021	Multicentre IT, PL, GR, UK	Multicentre retrospective	23 years	1997–2020	334	134	80	20	2/20	END vs. no-END
Dell’Aversana, 2021	Naples, IT	Retrospective	15 years	1999–2014	119	58	16	8	3/8	END vs. no-END
Fang, 2019	Zhenzhou, CN	Retrospective	14 years	2000–2014	144	52	144	52	15/52	END vs. TND
Grasl, 2019	Vienna, AT | Toronto, CA	Multicentre retrospective	28 years	1989–2017	66	27	66	27	4/27	END vs. TND vs. no-END
Ketterer, 2019	Freiburg, DE	Retrospective	13 years	2003–2016	102	69	11	7	1/7	END vs. no-END
Lau, 2014	Sacramento, US	Retrospective	14 years	1999–2013	119	119	10	10	0/10	No comparator
Ali, 2014	New York, US	Retrospective	24 years	1985–2009	263	74	34	9	3/9	END vs. TND vs. no-END
Zbären, 2005	Berne, CH	Retrospective	12 years	1990–2002	83	41	16	7	0/7	END vs. no-END
Frankenthaler, 1993	Houston, US	Retrospective	25 years	1960–1985	99	99	12	12	0/12	No comparator

Abbreviations: *AciCC* acinic cell carcinoma, *END *elective neck dissection, *MRND *modified radical neck dissection, *TND* therapeutic neck dissection, *pN +* pathologically node positive

**Table 2:** Classification of data as AciCC-specific or pooled, by outcome

Study	Cohort	Parotid AciCC OLNM rate	OLNM by T-stage	OLNM by grade	Survival by T-stage	Survival by grade	Effect of END on survival
Ning, 2025	Mixed salivary	22.2%	Pooled	—	—	Pooled	Improved PFS in low/moderate grade; no OS benefit. Not AciCC specific
Vedula, 2023	AciCC (parotid)	7.4%	—	AciCC: Low − 3.5% Intermediate − 9.8% High − 32.4%	—	END AciCC OS: Low – 93.5% Intermediate – 82.6% High – 20.4%	END AciCC compared to non-END AciCC showed no survival difference (ns)
Kapsalas, 2023	Mixed salivary	10.6%	Pooled	—	—	—	Not assessed
van Weert, 2022	AciCC-only (incl. non-parotid)	0%	—	—	AciCC (adv stage) RFS HR 9.7 DSS HR 13.7 OS HR 7.0 (all *p* < 0.001)	AciCC (HGT) RFS HR 2.9 DSS HR 3.2 OS HR 2.0 (all *p* ≤ 0.002)	Not assessed
Fussey, 2021	Mixed salivary‡	9.5%	—	—	AciCC OS: T1–2 96% T3–4 78% AciCC RFS: T1–2 93%, T3–4 67%	AciCC OS: low grade 100% high grade 77% AciCC RFS: low grade 96% high grade 69%	AciCC: no benefit OS 95% vs. 90% DFS 88% vs. 85% RFS 90% vs. 90%
Dell’Aversana, 2021	Mixed salivary	37.5%	Pooled	—	—	—	Pooled
Fang, 2019	AciCC-only (parotid)	28.8%	—	—	AciCC (DMC) T3–4 worse univariate *p* = 0.004 (HR 2.67, ns)	AciCC (DMC) HGT HR 4.22 (1.95–15.55) *p* < 0.001	Not assessed
Grasl, 2019	AciCC-only (parotid)	14.8%	AciCC T1–2: 7.3% T3–4: 18.2% (ns)	AciCC Low: 7.3% Int/high: 11.1% (ns)	AciCC OS ns (*p* = 0.126) DSS ns (*p* = 0.505) DFS ns (*p* = 0.329)	AciCC (G1 vs. G2–3) OS 96 vs. 69% (ns) DSS 100 vs. 69% (*p* = 0.007) DFS 93 vs. 49% (*p* = 0.002)	AciCC: no sig benefit OS 97 vs. 92% (ns) DSS 100 vs. 96% (ns) DFS 96 vs. 87% (ns)
Ketterer, 2019	Mixed salivary	14.3%	—	—	Pooled	Pooled	Pooled: no benefit
Lau, 2014	Mixed salivary	0%	AciCC (0%)	—	—	—	Not assessed
Ali, 2014	Mixed salivary	33.3%	—	—	—	Pooled	Pooled
Zbären, 2005	Mixed salivary	0%	—	—	—	—	Pooled
Frankenthaler, 1993	Mixed salivary	0%	—	AciCC (0%)	—	—	Not assessed

Abbreviations: *AciCC* acinic cell carcinoma specific data, *OLNM* occult lymph node metastasis, *END* elective neck dissection, *RFS* recurrence free survival, *PFS* progression free survival, *OS* overall survival, *HGT* high-grade transformation, *ns*  not statistically significant, *DMC* distant metastasis control, *DSS* disease-specific survival, *HR* hazard ratio

Legend: –=not reported; not assessed=survival as an outcome examined but not for END specifically; Pooled=reported across mixed salivary histology ‡ Fussey et al. is a mixed-salivary study; AciCC-specific data were derived from the authors’ patient-level raw dataset, provided on request. Grade- and T-stage-stratified survival are descriptive, given small subgroup numbers

For disease- and recurrence-free survival, Fussey et al. [9] reported the AciCC END group had a DFS of 85% and RFS of 90%, while the local excision group showed a slightly better DFS of 88% and an identical RFS of 90% [9]. Dell’Aversana Orabona et al. [10] noted that none of the AciCC patients who underwent END experienced recurrence during follow-up; however, AciCC patients without END in that same study also remained recurrence-free. Zbären et al. [13] reported a single AciCC patient who did not receive END, developed local and neck recurrence at 55 months, however this patient did not receive adjuvant radiotherapy. Other studies did not report on DFS or RFS directly. This indicates AciCC has some potential for metastasis and recurrence, even though formal survival rates like RFS or DFS are not consistently reported across studies.

Table 2 additionally demonstrates variable reported OLNM rates for AciCC ranging from 0% to 37.5%. The weighted mean of OLNM was 9.8% and median 10.6%. This weighted mean was derived as the total number of AciCC patients with pathologically positive nodes (pN+) divided by the total number of AciCC patients undergoing END across the included studies (Table 1), so that each study contributed in proportion to its number of dissected AciCC necks. Therefore, this value is presented as a descriptive crude pooled proportion. Formal statistical pooling and significance tests were not performed due to the marked heterogeneity between studies.

Recurrence occurred in both END and non-END groups across studies, but with no consistent pattern favouring one approach. Several studies lacked histology-specific recurrence data, limiting AciCC conclusions. The prognostic significance of T-stage in AciCCs has been inconsistently reported across the literature.

With respect to T-stage and OLNM, Lau et al. [14] reported no OLNM among 10 AciCC patients, despite inclusion of tumours ranging from T1 to T4. Kapsalas et al. [15] reported AciCC OLNM across stages T1–T3. However, their statistical analysis of T-stage and nodal involvement (χ² = 0.719, *p* = 0.396) was performed across the whole low-grade parotid cancer cohort rather than for AciCC specifically. Grasl et al. [4] found OLNM in 7.3% of T1–2 parotid AciCC compared with 18.2% of T3–4 tumours, though this difference was not statistically significant (*p* = 0.087). In contrast, Ning et al. [12] observed a higher OLNM rate in all types of T1–2 parotid cancers (27.9%) than in T3–4 cancers (14.3%), although this association was again not statistically significant (*p* = 0.448).

With respect to T-stage and survival outcomes, Grasl et al. [4] reported no significant differences in OS (*p* = 0.126), DSS (*p* = 0.505), or DFS (*p* = 0.329) between T1–2 and T3–4 AciCC tumours. In contrast, Ketterer et al. [11], although not stratifying by histology, found significantly worse OS (*p* = 0.001) and DFS (*p* = 0.003) in patients with parotid malignancies staged T3 or higher. Dell’Aversana Orabona et al. [10] also reported higher OLNM rates in T3 (18%) and T4 (41%) parotid tumours compared to T1 (0%) and T2 (8%) across their cohort, suggesting a relationship between advanced T-stage and nodal spread. Stronger evidence comes from van Weert et al. [16], who demonstrated that advanced-stage AciCC (stage III–IV, predominantly T3–4) was associated with significantly worse OS (hazard ratio [HR] 7.0, *p* < 0.001), DSS (HR 13.7, *p* < 0.001), and RFS (HR 9.7, *p* < 0.001). Fang et al. [17] similarly found that T3–4 parotid AciCC was associated with poorer distant metastasis control (*p* = 0.004), although T-stage was not independently predictive in multivariate analysis (HR 2.674, *p* = 0.089).

Two studies stratified AciCC data and compared the OLNM rate by histological grade [4, 18]. Grasl et al. [4] showed that AciCCs with HGT had an OLNM rate of 11.1% compared to low-grade tumours at 7.3%. With a relatively small sample size, these values were not statistically significant (*p* = 0.871). Vedula et al. [18] similarly reported, in AciCC specifically, that OLNM increased with poorer differentiation (well 3.5%, moderate 9.8%, poorly differentiated 32.4%).

Eight of thirteen included studies evaluated tumour grade in relation to survival outcomes. Grasl et al. [4], Fussey et al. [9], Fang et al. [17], Van Weert et al. [16] and Vedula et al. [18] reported grade-related survival specifically for AciCC, while the remaining studies that evaluated grade did so across mixed salivary histology or did not report on it at all. In the AciCC-specific subset of Fussey et al. [9], derived from their patient-level raw data, survival declined with higher grade (5-year OS 100% in low-grade vs. 77% in high-grade tumours) and with advanced T-stage (96% in T1–2 vs. 78% in T3–4). Their published, histology-pooled multivariable analysis likewise found HGT independently associated with worse OS (HR = 2.70, *p* = 0.005). Van Weert et al. [16] reported that high-grade transformation (HGT) in AciCC was associated with worse OS (HR = 2.0, *p* = 0.002), DSS (HR = 3.2, *p* = 0.001), and RFS (HR = 2.9, *p* < 0.001). Vedula et al. [18] reported END specific OS stratified by tumour grades, and showed that survival declined with poorer differentiation (well 93.5%, intermediate 82.6% to poorly differentiated 20.4%). Within each grade, END conferred no significant survival benefit over non-END (well *p* = 0.709, moderate *p* = 0.284, poor *p* = 0.117). Grasl et al. [4] likewise found, in parotid AciCC, that intermediate/high-grade tumours had significantly worse 5-year DSS (100% vs. 69%, *p* = 0.007) and DFS (93% vs. 49%, *p* = 0.002), with high-grade an independent predictor of DFS (HR 8.62, *p* = 0.010), although the effect on OS was not significant (96% vs. 69%, *p* = 0.148). Fang et al. [17] reported that HGT independently predicted poorer distant metastasis control in parotid AciCC (HR 4.22, 95% CI 1.95–15.55, *p* < 0.001).

In regard to the pooled histological data, Ketterer et al. [11] found improved OS (*p* = 0.018) and DFS (*p* = 0.019) in low-grade tumours. Ning et al. [12] found no significant differences in progression-free survival (PFS) or OS between the non-END and END groups for patients with low-grade or well-differentiated tumours (*p* > 0.05) [12]. Though END was associated with significantly prolonged PFS in patients with tumours of higher grade or low to moderate differentiation (*p* < 0.05). Ali et al. [19] reported poorer 5-year neck recurrence-free survival (RFS) in pathologically node positive patients treated with END compared with pN0 patients (80.5% vs. 97.3%, *p* = 0.05), with recurrences associated with high-grade histology.

The methodological quality of included studies, as assessed using the NOS tool (Table 3), was good overall [8]. All thirteen included studies achieved the maximum of four stars in the ‘selection’ domain, reflecting representative cohorts and reliable ascertainment of exposure. Performance in the ‘comparability’ domain was more variable. While most studies scored the maximum two stars, five studies (Zbären [13], Ali [19], Ketterer [11], Grasl [4], and Kapsalas [15]) received only one star due to limited adjustment for confounders, and van Weert [16] scored zero as no adjustment was attempted. In the ‘outcome’ domain, most studies scored the maximum three stars. Lau et al. received only one star due to absent follow-up reporting, while Zbären [13] and Frankenthaler [20] scored two stars, each lacking clear reporting of loss to follow-up. Overall, all studies were classified as ‘good quality’ under the NOS thresholds, though the main methodological limitations arose from inadequate adjustment for confounders and incomplete outcome reporting [8].

**Table 3:** Newcastle–Ottawa scale risk of bias assessment

Study	Selection (1ea)				Comparability (max 2)	Outcome (1ea)			Quality score
	Representativeness of the intervention cohort	Selection of the non-intervention cohort	Ascertainment of the intervention	Demonstration the outcome of interest wasn’t present at study start	Comparability of cohorts on the basis of design/analysis	Assessment of outcome	Adequate follow up time	Adequacy of follow up	
Ning, 2025	★	★	★	★	★★	★	★	★	9
Vedula, 2023	★	★	★	★	★★	★	★	★	9
Kapsalas, 2023	★	★	★	★	★☆	★	★	★	8
van Weert, 2022	★	★	★	★	☆☆	★	★	★	7
Fussey, 2021	★	★	★	★	★★	★	★	★	9
Dell’Aversana, 2021	★	★	★	★	★★	★	★	★	9
Fang, 2019	★	★	★	★	★★	★	★	★	9
Grasl, 2019	★	★	★	★	★☆	★	★	★	8
Ketterer, 2019	★	★	★	★	★☆	★	★	★	8
Lau, 2014	★	★	★	★	★★	★	☆	☆	7
Ali, 2014	★	★	★	★	★☆	★	★	☆	7
Zbären, 2005	★	★	★	★	★☆	★	★	★	8
Frankenthaler, 1993	★	★	★	★	★★	★	★	☆	8

## Discussion

Considerable heterogeneity was observed in the reporting of study outcomes. While all included studies reported rates of OLNM in cN0 parotid AciCC, the data were frequently pooled with other parotid malignancy subtypes. As a result, detailed analyses of recurrence, survival, or tumour-specific stratification by T-stage or histological grade were unable to be extracted. The choice of comparator group also varied, with some studies comparing END to primary surgery without neck dissection, and others comparing END to TND in clinically node positive patients. Reporting of follow-up was variable; most studies reported crude event rates at last follow-up, with few providing time-to-event or stratified survival analyses.

### The effect of elective neck dissection

While the survival benefit of END in cN0 AciCC remains uncertain, management decisions should not rely solely on the raw OLNM rates. These rates are influenced by tumour-related factors such as advanced T-stage and HGT, which themselves are markers of more aggressive disease. Moreover, because END is preferentially offered to patients with these higher-risk features, comparisons between END and non-END groups are confounded by indication; any apparent benefit of END may reflect differences in underlying disease severity rather than a true effect of the procedure itself. There is a lack of strong evidence supporting routine END in terms of improving OS and DFS, and the potential risks, including stroke, shoulder dysfunction, neck paraesthesia and cosmetic concerns, should be carefully weighed against potential benefits [9, 21].

Elective neck irradiation (ENI) represents an alternative, non-surgical strategy for nodal control in the cN0 neck. Lau et al. [14] argue against a meaningful role for it, as none of their 10 AciCC patients spanning T1-T4, harboured OLNM, and further reported no neck relapses in AciCC irrespective of whether ENI was administered. This is consistent with the low OLNM burden for AciCC observed throughout the present review. A formal comparison between ENI and END nonetheless lies beyond the scope of this review and warrants dedicated study.

Furthermore, variability in END definitions, surgical techniques, inconsistent outcome reporting, pooled histological data, and adjuvant treatment practices across institutions make it difficult to isolate its specific impact in AciCC. Importantly, there is currently no evidence-based OLNM threshold that mandates the use of END in this tumour type. The often-cited 20% benchmark for performing elective neck dissection originates from studies in oral squamous cell carcinoma (SCC), particularly Weiss et al. [22], and has been widely adopted despite its empirical and disease-specific basis. Moreover, the 20% figure is itself a decision-analytic break-even point rather than a biological constant. It is contingent on the modelled assumptions of nodal-dissection morbidity, surveillance reliability, and salvage feasibility, and shifts as these inputs change [22]. Given the distinct biological behaviour of AciCC compared to SCCs, direct extrapolation of this threshold must be cautioned. Contemporary practice for other head and neck malignancy has correspondingly moved towards risk-adapted neck management, in which the decision integrates not only the estimated OLNM rate but also tumour-specific risk modifiers like HGT and advanced T-stage, and patient- and system-level factors including surgical morbidity, follow-up, and institutional resources [23]. Such individualised frameworks have been advocated at other head and neck sites [23] and may translate well for AciCC. Collectively, these factors underscore the need for prospective, histology-specific research to determine a more rational and evidence-based approach to elective neck management in AciCC.

This highlights a need for more robust research in this area. Patient-centred considerations including age, comorbidities, cosmetic impact, and overall goals of care must also be weighed in multi-disciplinary treatment planning. Given the heterogeneity of existing studies, variable surgical approaches, and confounding from adjuvant therapies, the role of END in AciCC should be interpreted within the broader clinical and pathological context.

This risk-adapted approach is consistent with the wider parotid malignancy literature. In a pooled patient-level analysis of parotid basal cell adenocarcinoma, Chiari et al. [24] reported similarly low rates of nodal metastasis (approximately 6% clinical and pathological) and likewise concluded that routine END is not warranted in a cN0 neck. Conversely, in cutaneous metastatic neck and parotid SCC, where OLNM rates are considerably higher (24–42%) and extranodal extension strongly predicts poorer survival, elective neck treatment demonstrated a more justifiable role [25]. Together, these observations underscore that the threshold for END should track the OLNM risk of the specific entity, which in parotid AciCC remains low.

### The effect of T-stage

The role of T-stage as a prognostic factor in AciCC remains contested, with evidence drawn from a mixture of small institutional cohorts and larger population-based studies. Early series papers such as by Grasl et al. [4] and Kapsalas et al. [15] did not determine T-stage as a potential predictor of OLNM or survival outcomes. However, larger-scale analyses, most notably by van Weert et al. [16], have found an association between advanced T-stage and poorer OS, DSS and RFS. This finding, derived largely from a single large AciCC-specific cohort, lends some support to T-stage as a prognostic factor. Notably however, Fang et al. [17] did not find T-stage to be independently predictive on multivariate analysis (HR 2.674, *p* = 0.089), tempering its role as an independent prognostic factor.

Taken together, these findings highlight a diversity and divergence in the literature. Smaller single-centre series, often underpowered, tend to show no strong relationship between T-stage and outcomes, while the larger contemporary AciCC-specific cohort of Van Weert et al. [16] suggests that advanced T-stage is linked with poorer prognosis. This discrepancy likely reflects both the rarity of AciCC and the methodological limitations of older studies. What emerges most clearly is that while T-stage should not be used in isolation, advanced T-stage may identify patients at increased risk of poor survival. However, as this rests largely on a single cohort and is subject to selection bias, it should inform rather than dictate management, and any consideration of more aggressive strategies such as END must be weighed against the absence of a demonstrated survival benefit.

### The effect of tumour grade

Tumour grade is an important prognostic factor in salivary gland malignancies. Across the included studies, higher tumour grade correlated with poorer survival outcomes, though the strength of association and statistical significance varied.

Van Weert et al. [16], who stratified for AciCCs, demonstrated a clear negative impact of HGT on OS, DSS, and RFS, demonstrating that poorer differentiation represents a more aggressive nature with worse clinical outcomes. This finding was supported by Vedula et al. [18] who also found a trend of poorer differentiation with decreased OS, though their findings were not statistically significant. These findings are consistent with Price et al. [26] who showed a decrease in both 5-year OS and RFS in AciCC-HGT, concluding that HGT should be considered when determining indications for treatment options. Chatelet et al. [27] also found that AciCC-HGT was associated with worse DFS (*p* < 0.001). However, these are general survival outcome findings by Price and Chatelet and not stratified to an OLNM cohort. When pooled across histological subtypes, the effect of tumour grade on survival outcomes was reproduced. Ketterer et al. [11] showed that higher grade tumours were associated with worse OS and DFS.

Metastatic outcomes were also associated with tumour grade. Grasl et al. [4] and Vedula et al. [18] reported numerically higher rates of OLNM in AciCCs with HGT than in low- and intermediate-grade tumours, although these differences were based on small sample size and did not reach statistical significance. Fang et al. [17] also demonstrated a significant reduction in distant metastasis control in HGT-AciCC, while Van Weert et al. [16] confirmed a higher incidence of nodal metastases and aggressive histological features (high mitotic rate in 89% and extra-glandular growth in 84%) in this subgroup. The predominance of HGT among patients requiring TNDs further highlights the metastatic potential of poorly differentiated tumours. In contrast, Frankenthaler et al. [20] reported no OLNM in AciCC regardless of grade, though data were not extrapolated to consider associated survival outcomes.

These grade-dependent associations must, however, be interpreted considering the well-documented diagnostic difficulty of salivary gland pathology. In a recent national survey and expert-review series, salivary gland tumours were among the most frequently referred for second opinion and showed high rates of major diagnostic discrepancy, with revisions in grade or even benign-versus-malignant status materially altering management [28]. As accurate determination of HGT and tumour grade is central to the risk stratification discussed above, reproducible histopathological classification, ideally with expert or centralised review, is a prerequisite that cannot be overlooked for reliable grade-based decisions on elective neck management in AciCC.

The literature demonstrates that END may not be routinely beneficial in low-grade, well-differentiated AciCC. Particularly in early T-stage tumours, these patients generally have good outcomes with local excision alone, demonstrated by multiple retrospective studies showing low rates of OLNM as well as similar OS and DFS rates [9, 14, 20]. From a pathophysiological perspective, well-differentiated AciCCs retain more cellular organisation resembling normal salivary acinar cells, with better-defined cell boundaries and more organised growth patterns that limit invasive and metastatic potential [29]. Conversely, high-grade or poorly differentiated AciCC and advanced T-stage tumours show higher OLNM. However, the suggestion that END can be meaningfully beneficial in these patients is difficult to make as it is hindered by the heterogeneity of data supporting it.

### Limitations & future recommendations

Interpretation of the current evidence is limited by several factors. Most studies of AciCC are retrospective, and small, varying studies provide histology-specific analyses stratified to either T-stage or tumour grade. Many series are underpowered due to the rarity of the disease, reducing the ability to detect statistically significant associations. There is also heterogeneity in how several outcomes were reported, how neck management was approached, and whether survival endpoints were measured consistently across cohorts. Furthermore, some large database studies do not break down data analysis by histology, making it difficult to draw firm conclusions specific to AciCC. These limitations highlight the need for collaborative, multi-institutional studies with a sufficient sample of AciCC patients and uniform reporting standards to clarify the prognostic role of T-stage and to guide evidence-based decisions around neck management. In addition, the certainty of the evidence was not formally graded, and publication bias could not be reliably assessed given the small number of comparable studies per outcome; these represent further limitations.

In addition to the surgical management of AciCC, it highlights a broader methodological challenge inherent to the study of rare pathologies. The limited number of cases, pooling of histological diagnoses, and inconsistent reporting across institutions make it difficult to perform robust analyses or generate high-level evidence. These limitations are not unique to AciCC, but reflect a wider issue in rare disease research, where traditional systematic review methodologies may be constrained by fragmented and underpowered datasets.

In this context, there is increasing value in the development of multi-institutional registries and collaborative databases that can prospectively capture standardised variables and outcomes. Such platforms may allow for larger datasets with increased homogeneity, enabling more reliable evaluation of treatment strategies and prognostic factors over time. As data infrastructure continues to evolve, these approaches may complement and perhaps bear potential to supersede traditional evidence synthesis methods in rare disease research.

## Conclusion

END does not appear to confer a consistent survival benefit in low-grade, early-stage AciCC, where outcomes with local excision alone are generally favourable. HGT emerges as an adverse prognostic factor associated with increased risk of recurrence and metastatic potential, whereas the independent prognostic value of advanced T-stage is less certain, resting largely on individual retrospective cohorts. In these higher-risk subgroups, END has been proposed as a means of improving regional control, and one study derived from a mixed-histology parotid cohort reported improved progression-free survival in higher-grade tumours [12]. However, these findings did not consistently translate into an overall survival benefit. Crucially, because END is preferentially performed in patients with more aggressive disease, these comparisons are confounded by indication and cannot establish a causal therapeutic benefit. Its role in higher-risk subgroups therefore remains uncertain, pending prospective, adequately controlled studies.

The findings also suggest that tumour biology may be a more important determinant of outcome than OLNM alone [10, 16]. More broadly, the limitations encountered in this review reflect the challenges of studying rare malignancies using conventional evidence synthesis methods. Future progress will require collaborative, multi-institutional registries with standardised data collection, enabling more robust evaluation of treatment strategies and better identification of patient subgroups.

## Protocol differences

This review departed from the registered protocol in some respects. Methodological quality was appraised using the Newcastle–Ottawa Scale rather than the pre-specified MINORS tool, as all included studies were observational cohort designs for which the Newcastle–Ottawa Scale is the validated instrument. Owing to the marked heterogeneity and the small number of comparable studies per outcome, the planned meta-analysis was not performed. Similarly, formal assessment of publication or small-study bias was not undertaken, as these methods are unreliable when few studies contribute to an outcome. A formal GRADE assessment of the certainty of evidence was not performed; this is acknowledged as a limitation.

## Supplementary Information

Below is the link to the electronic supplementary material.

Supplementary Material 1 (DOCX 27.6 KB)

## Data Availability

The data that support the findings of this study are available from the corresponding author upon reasonable request.
