# Supplementary material for: The role of elective neck dissection in parotid acinic cell carcinoma: a systematic review
Source: Oral Maxillofac Surg. 2026 Aug 26;30(1):140. doi: 10.1007/s10006-026-01633-6 (PMC13506562; doi:10.1007/s10006-026-01633-6)
Supplement: Supplementary file 1 [file 10006_2026_1633_MOESM1_ESM.docx]

**Supplementary Material 1 — Electronic Search Strategies**

*Manuscript: The role of elective neck dissection in parotid acinic cell carcinoma — A Systematic Review.*

Databases searched: PubMed (MEDLINE; 1946–present), EMBASE (1974–present), and CINAHL (1981–present). Searches combined controlled vocabulary (MeSH / EMTREE / CINAHL Headings) with free-text keywords. Search last run: 7 May 2025. Strategies are reproduced as registered in the PROSPERO protocol (CRD420251046849).

**1. PubMed (MEDLINE)**

((((("salivary glands"[MeSH Terms] OR "Parotid Gland"[MeSH Terms] OR parotid* OR parotid gland OR salivary gland*)
AND
("carcinoma, acinar cell"[MeSH Terms] OR "Parotid Neoplasms"[MeSH Terms] OR parotid acinic cell carcinoma* OR parotid ACC OR salivary gland acinic cell carcinoma* OR salivary gland ACC OR acinic cell carcinoma OR AciCC*))
AND
("Neoplasm Staging"[MeSH] OR stag* OR grade OR "tumor grade" OR "tumour grade" OR "tumor stag*" OR "tumour stag*" OR "T stag*" OR "T-stag*" OR "tumor classification*" OR "tumour classification*" OR "tumor grade*" OR "tumour grade*" OR "cancer stag*" OR "staging system*" OR "tumor progression" OR "tumour progression" OR "pathological stag*" OR "clinical stag*" OR "neoplasm stag*" OR "cancer progression" OR "T"))
AND
("neck dissection"[MeSH Terms] OR ("neck" AND "dissect*") OR "neck dissection" OR "elect* neck dissect*" OR ("elect*" AND "neck" AND "dissect*") OR "prophylact* neck dissect*" OR ("prophylact*" AND "neck" AND "dissect*") OR "prevent* neck dissect*" OR ("prevent*" AND "neck" AND "dissect*") OR "cervical neck dissect*" OR ("cervical*" AND "neck" AND "dissect*") OR "lymph node neck dissect*" OR ("lymph node*" AND "neck" AND "dissect*") OR "lymph node dissect*" OR ("lymph node" AND "dissect*") OR "nodal dissect*" OR ("nodal" AND "dissect*") OR "node dissect*" OR ("node" AND "dissect*") OR "lymph node excision"[MeSH Terms] OR ("lymph node" AND "excis*") OR "node excis*" OR ("node" AND "excis*") OR "nodal excis*" OR ("nodal" AND "excis*") OR "lymphadenectom*"))
AND
(excis* OR surgery OR resect* OR remov* OR (local AND excis*) OR (local AND surgery) OR (local AND resect*) OR (local AND remov*) OR (parotid AND excis*) OR (parotid AND surgery) OR (parotid AND resect*) OR (parotid AND remov*) OR "parotidectom*" OR ("salivary gland" AND excis*) OR ("salivary gland" AND surgery) OR ("salivary gland" AND resect*) OR (salivary AND remov*) OR (salivary AND excis*) OR (salivary AND surgery) OR (salivary AND resect*) OR (salivary AND remov*) OR (gland AND excis*) OR (gland AND surgery) OR (gland AND resect*) OR (gland AND remov*) OR (tumor AND excis*) OR (tumor AND surgery) OR (tumor AND resect*) OR (tumor AND remov*) OR (tumour AND excis*) OR (tumour AND surgery) OR (tumour AND resect*) OR (tumour AND remov*)))
AND
("lymph nodes"[MeSH Terms] OR "Neoplasm Metastasis"[MeSH] OR "Lymphatic Metastasis"[MeSH] OR "occult lymph node metastas*" OR "nodal metastas*" OR "node metastas*" OR "lymph node metastas*" OR "lymph node involvement*" OR "lymphatic spread*" OR "regional metastas*" OR "occult metastas*" OR "micrometastases" OR "cervical lymph node metastas*" OR "cervical node metastas*" OR "cervical nodal metastas*" OR "cervical metastas*" OR "lymphatic dissem*" OR nodal OR node)

**2. EMBASE**

((((('salivary gland'/exp OR 'Parotid Gland'/exp OR parotid* OR 'parotid gland' OR 'salivary gland*')
AND
('acinar cell carcinoma'/exp OR 'parotid gland tumor'/exp OR 'parotid acinic cell carcinoma*' OR 'parotid ACC' OR 'salivary gland acinic cell carcinoma*' OR 'salivary gland ACC' OR 'acinic cell carcinoma' OR AciCC*))
AND
('cancer staging'/exp OR stag* OR grade OR 'tumor grade' OR 'tumour grade' OR 'tumor stag*' OR 'tumour stag*' OR 'T stag*' OR T-stag* OR 'tumor classification*' OR 'tumour classification*' OR 'tumor grade*' OR 'tumour grade*' OR 'cancer stag*' OR 'staging system*' OR 'tumor progression' OR 'tumour progression' OR 'pathological stag*' OR 'clinical stag*' OR 'neoplasm stag*' OR 'cancer progression' OR ‘T’))
AND
('neck dissection'/exp OR (neck AND dissect*) OR 'neck dissection' OR 'elect* neck dissect*' OR (elect* AND neck AND dissect*) OR 'prophylact* neck dissect*' OR (prophylact* AND neck AND dissect*) OR 'prevent* neck dissect*' OR (prevent* AND neck AND dissect*) OR 'cervical neck dissect*' OR (cervical* AND neck AND dissect*) OR 'lymph node neck dissect*' OR ('lymph node*' AND neck AND dissect*) OR 'lymph node dissect*' OR ('lymph node' AND dissect*) OR 'nodal dissect*' OR (nodal AND dissect*) OR 'node dissect*' OR (node AND dissect*) OR 'lymph node dissection'/exp OR ('lymph node' AND excis*) OR 'node excis*' OR (node AND excis*) OR 'nodal excis*' OR (nodal AND excis*) OR lymphadenectom*))
AND
(excis* OR surgery OR resect* OR remov* OR (local AND excis*) OR (local AND surgery) OR (local AND resect*) OR (local AND remov*) OR (parotid AND excis*) OR (parotid AND surgery) OR (parotid AND resect*) OR (parotid AND remov*) OR parotidectom* OR ('salivary gland' AND excis*) OR ('salivary gland' AND surgery) OR ('salivary gland' AND resect*) OR (salivary AND remov*) OR (salivary AND excis*) OR (salivary AND surgery) OR (salivary AND resect*) OR (salivary AND remov*) OR (gland AND excis*) OR (gland AND surgery) OR (gland AND resect*) OR (gland AND remov*) OR (tumor AND excis*) OR (tumor AND surgery) OR (tumor AND resect*) OR (tumor AND remov*) OR (tumour AND excis*) OR (tumour AND surgery) OR (tumour AND resect*) OR (tumour AND remov)))
AND
('lymph node'/exp OR 'metastasis'/exp OR 'lymph node metastasis'/exp OR 'occult lymph node metastas*' OR 'nodal metastas*' OR 'node metastas*' OR 'lymph node metastas*' OR 'lymph node involvement*' OR 'lymphatic spread*' OR 'regional metastas*' OR 'occult metastas*' OR micrometastases OR 'cervical lymph node metastas*' OR 'cervical node metastas*' OR 'cervical nodal metastas*' OR 'cervical metastas*' OR 'lymphatic dissem*' OR nodal OR node)

**3. CINAHL (via EBSCOhost)**

((((((MH "Salivary Glands+") OR (MH "Parotid Gland+") OR parotid* OR "parotid gland" OR "salivary gland*")
AND
((MH "Parotid Neoplasms+") OR "parotid acinic cell carcinoma*" OR "parotid ACC" OR "salivary gland acinic cell carcinoma*" OR "salivary gland ACC" OR "acinic cell carcinoma" OR AciCC*))
AND
((MH "Neoplasm Staging+") OR stag* OR grade OR "tumor grade" OR "tumour grade" OR "tumor stag*" OR "tumour stag*" OR "T stag*" OR T-stag* OR "tumor classification*" OR "tumour classification*" OR "tumor grade*" OR "tumour grade*" OR "cancer stag*" OR "staging system*" OR "tumor progression" OR "tumour progression" OR "pathological stag*" OR "clinical stag*" OR "neoplasm stag*" OR "cancer progression" OR T))
AND
((MH "Neck Dissection+") OR (neck AND dissect*) OR "neck dissection" OR "elect* neck dissect*" OR (elect* AND neck AND dissect*) OR "prophylact* neck dissect*" OR (prophylact* AND neck AND dissect*) OR "prevent* neck dissect*" OR (prevent* AND neck AND dissect*) OR "cervical neck dissect*" OR (cervical* AND neck AND dissect*) OR "lymph node neck dissect*" OR ("lymph node*" AND neck AND dissect*) OR "lymph node dissect*" OR ("lymph node" AND dissect*) OR "nodal dissect*" OR (nodal AND dissect*) OR "node dissect*" OR (node AND dissect*) OR (MH "Lymph Node Excision+") OR ("lymph node" AND excis*) OR "node excis*" OR (node AND excis*) OR "nodal excis*" OR (nodal AND excis*) OR lymphadenectom*))
AND
(excis* OR surgery OR resect* OR remov* OR (local AND excis*) OR (local AND surgery) OR (local AND resect*) OR (local AND remov*) OR (parotid AND excis*) OR (parotid AND surgery) OR (parotid AND resect*) OR (parotid AND remov*) OR parotidectom* OR ("salivary gland" AND excis*) OR ("salivary gland" AND surgery) OR ("salivary gland" AND resect*) OR (salivary AND remov*) OR (salivary AND excis*) OR (salivary AND surgery) OR (salivary AND resect*) OR (salivary AND remov*) OR (gland AND excis*) OR (gland AND surgery) OR (gland AND resect*) OR (gland AND remov*) OR (tumor AND excis*) OR (tumor AND surgery) OR (tumor AND resect*) OR (tumor AND remov*) OR (tumour AND excis*) OR (tumour AND surgery) OR (tumour AND resect*) OR (tumour AND remov*)))
AND
((MH "Lymph Nodes+") OR (MH "Neoplasm Metastasis+") OR (MH "Extranodal Extension+") OR "occult lymph node metastas*" OR "nodal metastas*" OR "node metastas*" OR "lymph node metastas*" OR "lymph node involvement*" OR "lymphatic spread*" OR "regional metastas*" OR "occult metastas*" OR micrometastases OR "cervical lymph node metastas*" OR "cervical node metastas*" OR "cervical nodal metastas*" OR "cervical metastas*" OR "lymphatic dissem*" OR nodal OR node)
